# Architectural Analysis for Novel Olive Crop Management

**DOI:** 10.3390/plants14111707

**Published:** 2025-06-03

**Authors:** Khouloud Annabi, Faouzi Haouala, AbdelKarim Hamrita, Rania Kouki, Foued Laabidi, Mokhtar Rejili, Samra Akef Bziouech, Mouna Mezghani Aïachi

**Affiliations:** 1 Research Laboratory of Agrobiodiversity and Ecotoxicology “LR21AGR02”, Higher Agronomic Institute of Chott Mariem, University of Sousse, Sousse 4002, Tunisia; 2 Department of Biology, College of Sciences, Imam Mohammad Ibn Saud Islamic University (IMSIU), Riyadh 11564, Saudi Arabia; 3 High Institute of Biotechnology of Monastir, University of Monastir, Monastir 5000, Tunisia; 4 Research Laboratory, Improving the Productivity of Olive Tree and Product Quality, Olive Tree Institute Sousse, Ibn Khaldoun Street, B.P.: 14, Sousse 4061, Tunisia; khouloud.annabi@gmail.com (F.L.); ayachiimouna@yahoo.fr (M.M.A.)

**Keywords:** architectural analysis, biometric characterisation, olives, cultivars, topographic mechanisation

## Abstract

Efficient fruit production, quality improvement, and timely harvesting are essential in olive cultivation, which requires optimised distribution and management of fruiting sites. This study aimed to support sustainable olive crop management by analysing the morphological characteristics of five cultivars (*Chemlali*, *Chetoui*, *Koroneiki*, *Meski*, and *Picholine*) under semi-arid Tunisian conditions. Through a detailed architectural analysis, we investigated the relationships between branching patterns, density, distribution of inflorescence and fruit sites, biometric traits (shoot length, internode number, and shoot dimensions), and geometric variability within each cultivar. Three trees per cultivar were analysed across three architectural units. The results showed marked architectural differences, highlighting the need for cultivar-specific strategies in planting, pruning, and orchard management. The distribution of shoots across botanical orders revealed unique branching patterns: *Chemlali* and *Koroneiki* showed thinner shoots and higher shoot density, reflecting strong apical dominance and their suitability for hyper-intensive systems. In addition, nonsignificant differences in long shoots’ insertion angles between *Meski*, *Chetoui*, and *Koroneiki* suggest compatibility for co-cultivation, facilitating mechanised maintenance and harvesting. Emphasis on inter-cultivar compatibility and architectural coherence is crucial for orchard design. These findings provide important insights for optimising orchard management practices to improve productivity, fruit quality, and operational efficiency.

## 1. Introduction

The Mediterranean basin encompasses nearly 97% of the global olive tree (*Olea europaea* L.) cultivation area, contributing to 96% of worldwide olive production [1]. This region holds significant economic sway within the global olive industry (96% of global olive production), emphasising the imperative for modernisation within olive farms [2]. Agricultural investments aimed at enhancing productivity include the adoption of efficient crop management practices, mechanisation of irrigation, pruning, and post-harvest management [2,3], as well as improved phytosanitary treatment. These advancements underscore the shift towards crossbreeding programmes, focusing on developing olive cultivars tailored for mechanisation, high yield, and resilience to environmental stresses [4].

Advancements in genomics and biotechnology have propelled plant breeding programmes forward, particularly through efficient genotyping methods [5,6]. However, the absence of efficient phenotyping techniques remains a bottleneck in varietal development [2]. Plant architecture analysis serves as a foundational discipline, facilitating a deeper comprehension of growth patterns, branching dynamics, and yield potential, thereby informing breeding strategies [7,8]. Conventional approaches to phenotypic data collection primarily focus on analysing trunk architecture to provide a simplified overview of a tree’s growth and developmental trajectory throughout its lifespan [9].

While numerous international studies have explored olive tree architectural development [10,11,12,13,14,15], limited attention has been paid to the interplay between trees or shoots within Tunisia [9,16]. Such analyses are critical for understanding vegetative architecture’s influence on reproductive processes and, consequently, the growth and fruiting strategies of different olive species and cultivars [7,17,18,19]. Improving the acquisition of plant traits such as morphological characteristics, flowering time, and yield has therefore become the main challenge limiting the design and prediction of the results of breeding programmes [13,20]. This aspect is particularly important in olive breeding due to the high genetic variability commonly obtained in seedling progeny [21].

Given this research gap, our study focuses on characterising the morphological traits of olive cultivars and their structural units over three consecutive growing years, with particular emphasis on the relationship between vegetative architecture and sexuality. We aim to provide insights into the morphological characteristics of the identified units and growth sites, facilitating the identification of future production sites within Tunisian olive orchards.

## 2. Results

### 2.1. Analysis of the Topographic Architectural Variability

#### 2.1.1. Evaluation of the Importance of Branching

**Figure 1:**
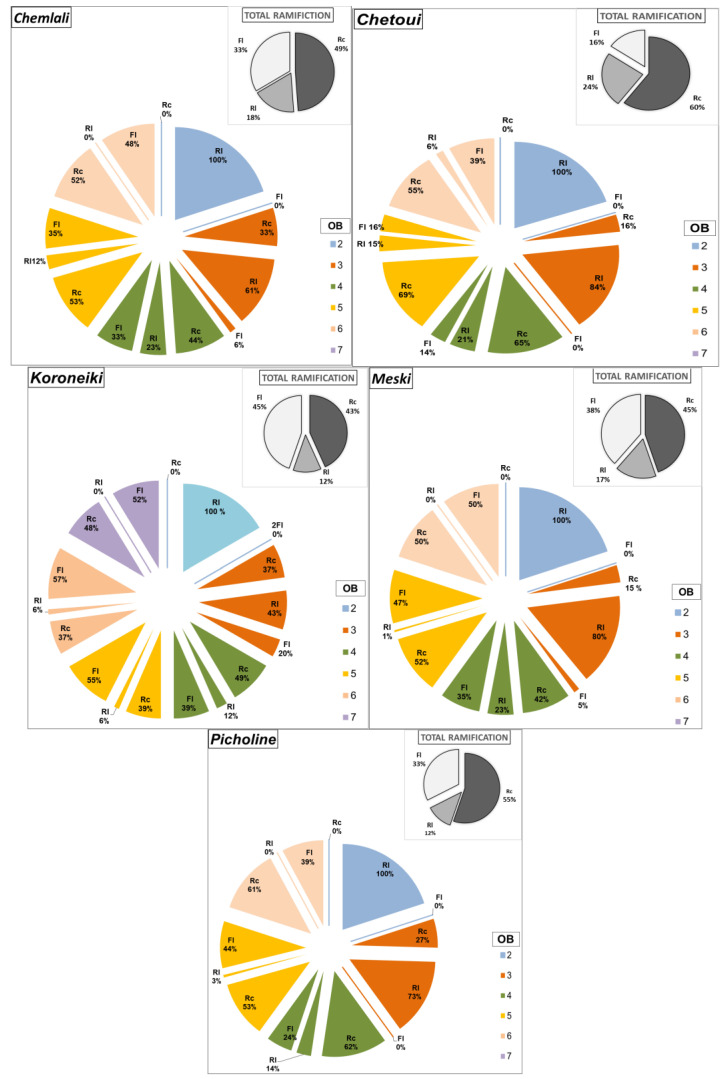
Distribution (in %) of the total branching by nature (Rc: short shoot; Rl: long shoot; FL: inflorescence) and botanical order (OB) per architectural unit of the cultivars *Chemlali*, *Chetoui*, *Meski*, *Koroneiki*, and *Picholine*.

Figure 1 shows the variation of the average percentage of developed shoots according to their type (long shoot, short shoot, or inflorescence) per architectural unit in each of the five studied cultivars (*Chemlali*, *Chetoui*, *Meski*, *Koroneiki*, and *Picholine*). The highest level of long-developed shoots was observed in the cultivar *Chetoui* (24%), followed by the cultivars *Chemlali* (18%), *Meski* (17%), *Picholine* (12%), and *Koroneiki* (12%) (Figure 1). At the scale of botanical orders, the highest percentages of long shoots concerning the total shoots developed in the same botanical order, for most of the cultivars, were located in the 3rd botanical order (OB3). It was about 84% for the cultivar *Chetoui*, 80% for the cultivar *Meski*, 61% for the cultivar *Chemlali*, and 43% for the cultivar *Koroneiki* (Figure 1).

The highest percentage of short shoots developed per architectural unit was observed in the cultivar *Chetoui* (60%), followed by the cultivars *Picholine* (55%), *Chemlali* (49%), *Meski* (45%), and *Koroneiki* (43%) (Figure 1). At the scale of botanical order (OB), the highest ratio of short shoots compared to the total shoots developed in the same botanical order (Rl, Rc, and Fl) was located in the 5th botanical order (OB5). It was about 69%, 53%, and 52% for the *Chetoui*, *Chemlali*, and *Meski* cultivars, respectively (Figure 1). On the other hand, the cultivars *Koroneiki* and *Picholine* had the highest percentage of short shoots compared to the total number of shoots developed in the 4th (49%) and 6th (62%) botanical orders, respectively (Figure 1). The cultivar *Koroneiki* produced the highest percentage of inflorescences per architectural unit (45%), followed by the cultivars *Meski* (38%), *Chemlali* (33%), *Picholine* (33%), and *Chetoui* (16%) (Figure 1). At the scale of botanical order (OB), the highest percentages of inflorescences compared to the total shoots developed in the same botanical order for the cultivars *Meski* (50%), *Chemlali* (48%), *Chetoui* (39%), and *Koroneiki* (57%) were in the 6th botanical order (OB6) (Figure 1).

#### 2.1.2. Estimation of Branching Density

**Figure 2:**
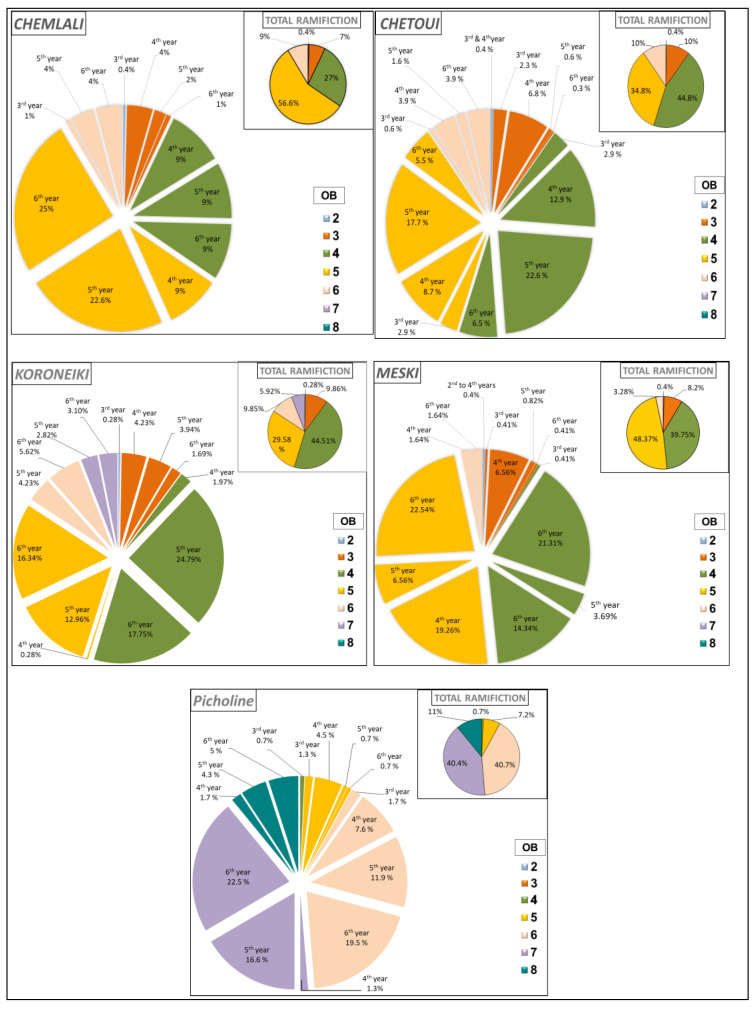
Variation of total branching (%) according to the botanical order (OB) and year of development (Year) per architectural unit of the cultivars *Chemlali*, *Chetoui*, *Meski*, *Koroneiki*, and *Picholine*.

Figure 2 shows the variation of the average percentage of developed shoots considering the botanical order and the year of growth of the shoot. The most important level of branching was marked in the OB5 for the cultivars *Meski* and *Chemlali*; it varied from 48.37% to 56.6% of the total developed shoots. For the cultivars *Chetoui* and *Koroneiki*, this percentage was about 44% and was mainly located in the 4th botanical order (OB4).

The lowest branching density for the five studied cultivars was marked in the second botanical order (OB2), with percentages varying from 0.28% (*Koroneiki*) to 0.4% (*Meski*). The highest branching density in the OB2 for the five studied cultivars was marked on the shoots developed in the 3rd year (2011) (Figure 2).

Regarding the branching density, the highest percentage in the OB3 was found at the level of shoots developed in the 4th year (2013) with consecutive percentages of 42.86% (*Koroneiki*), 61.11% (*Chemlali*), 80% (*Meski*), 67.74% (*Chetoui*) of the total shoots developed in the OB3 for each studied cultivar (Figure 2). The highest branching density in the OB4 was marked on the shoots developed either in the 4th year (2013), with consecutive percentages of 34.29% (*Chemlali*) and 53.61% (*Meski*), or in the 5th year (2014), with consecutive percentages of 55.70% (*Koroneiki*) and 50.36% (*Chetoui*) of the total shoots developed in the same botanical order (OB4) for each of the studied cultivars (Figure 2). In the OB5, the highest density of branching was found on the shoots developed either in the 4th year (2013), with consecutive percentages of 63.64% (*Picholine*) and 46.61% (*Meski*), or on the shoots developed in the 6th year (2015), with consecutive percentages of 55.24% (*Koroneiki*) and 43.92% (*Chemlali*) of the total shoots developed in the same botanical order (OB5) for each of the studied cultivars (Figure 2).

#### 2.1.3. Characterisation of the Shoots Carrying Architectural Units

**Figure 3:**
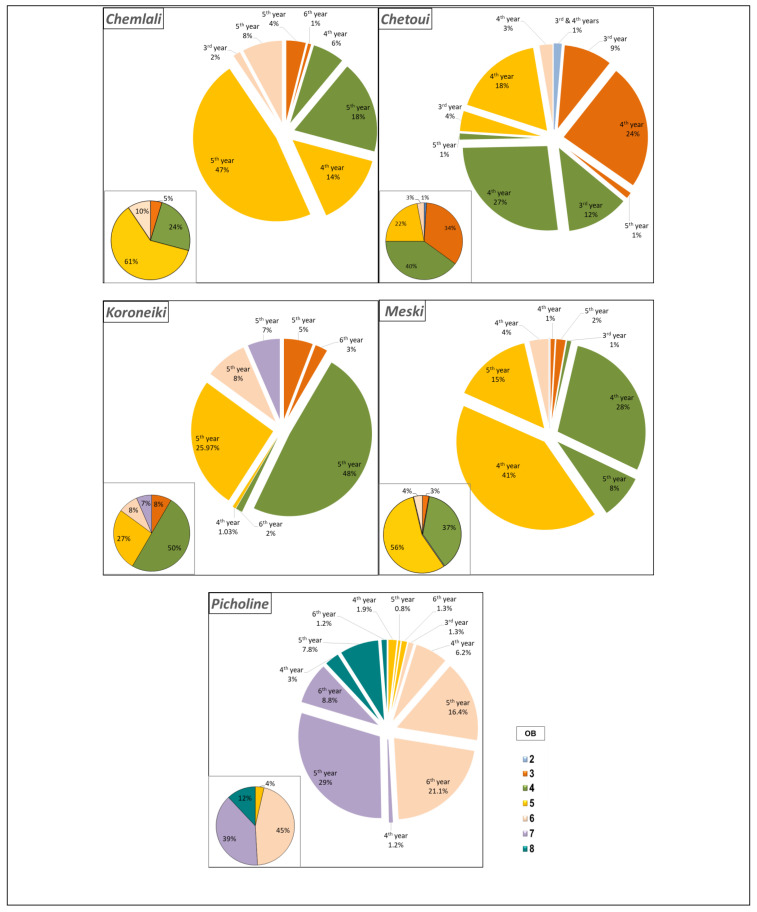
Distribution (%) of short shoots (Rc) according to the botanical order (OB) and year of development per architectural unit of the cultivars *Chemlali*, *Chetoui*, *Meski*, *Koroneiki*, and *Picholine*.

According to the architectural study carried out, all the studied cultivars tended to develop short shoots much more than long shoots or inflorescences. However, the difference between cultivars becomes clearer after comparing the percentage of each type of shoots according to the location by botanical order and according to the year of development of the shoots bearing each type of branch. The cultivars *Chemlali* (61%) and *Meski* (56%) produced the highest percentages of short shoots in the OB5 (Figure 3). The cultivars *Chetoui* (40%) and *Koroneiki* (50%) produced short shoots in the OB4 (Figure 3). Meanwhile, according to the year of development of bearing branches, the cultivars *Koroneiki*, *Picholine*, and *Chemlali* had the highest percentages of short shoots borne by shoots developed in the 5th year (2014), with 92.97%, 53.4%, and 77% of the total short shoots produced per architectural unit of each cultivar, respectively. The cultivars *Meski* and *Chetoui* had the highest percentages of short shoots supported by shoots developed in the 4th year (2013), with 74% and 72%, respectively (Figure 3).

**Figure 4:**
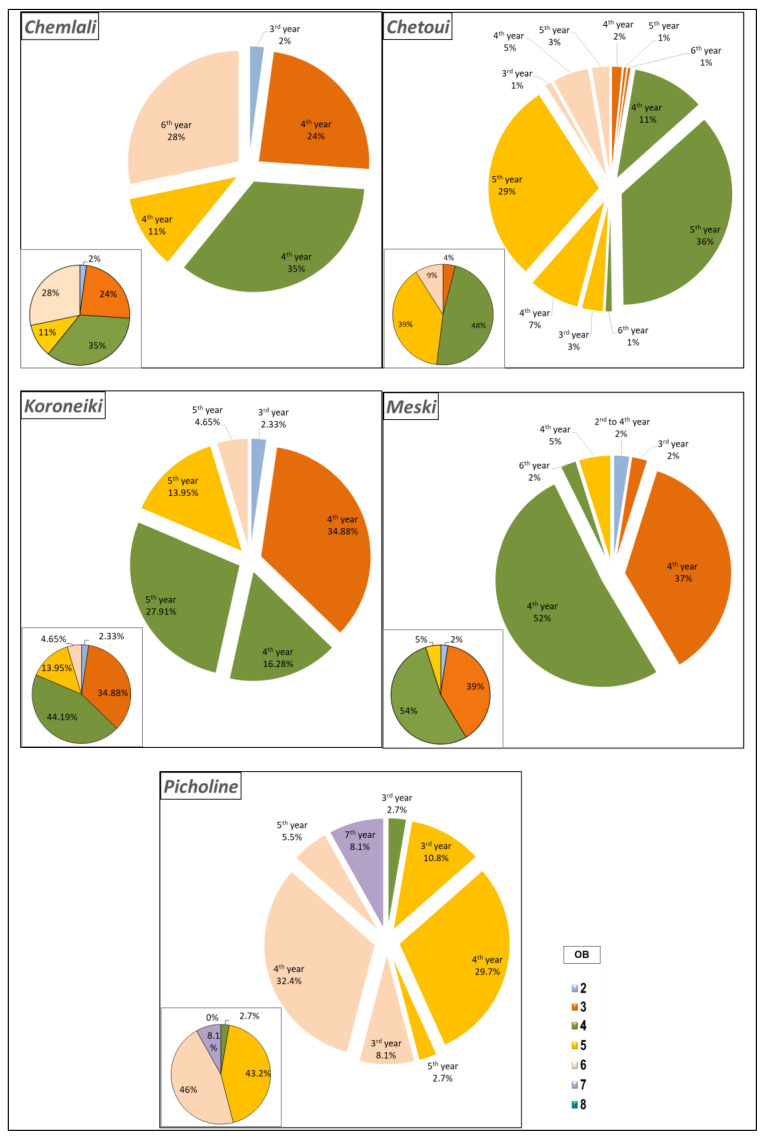
Distribution of long shoots (Rl) (%) according to the botanical order (OB) per architectural unit of the cultivars *Chemlali*, *Chetoui*, *Meski*, *Koroneiki*, and *Picholine*.

At the scale of architectural units, the highest percentages of long shoots were located in the OB4 of the cultivars *Koroneiki*, *Meski*, and *Chemlali*, with 44.19%, 54%, and 35%, respectively, of the total number of long shoots developed per architectural unit of each cultivar (Figure 4). For the cultivars *Chetoui* and *Picholine*, 48% and 46% of the total number of long shoots developed per architectural unit of each cultivar were located in the OB4 and the OB6, respectively (Figure 4). Regardless of the botanical order, the highest percentages of long shoots were carried by the shoots developed in the 4th year for the cultivars *Koroneiki* (51.16%), *Chemlali* (70%), *Picholine* (62.1%), and *Meski* (93%). The cultivar *Chetoui* produced 69% of long shoots at the level of developed bearing shoots in the 5th year (2014) (Figure 4).

**Figure 5:**
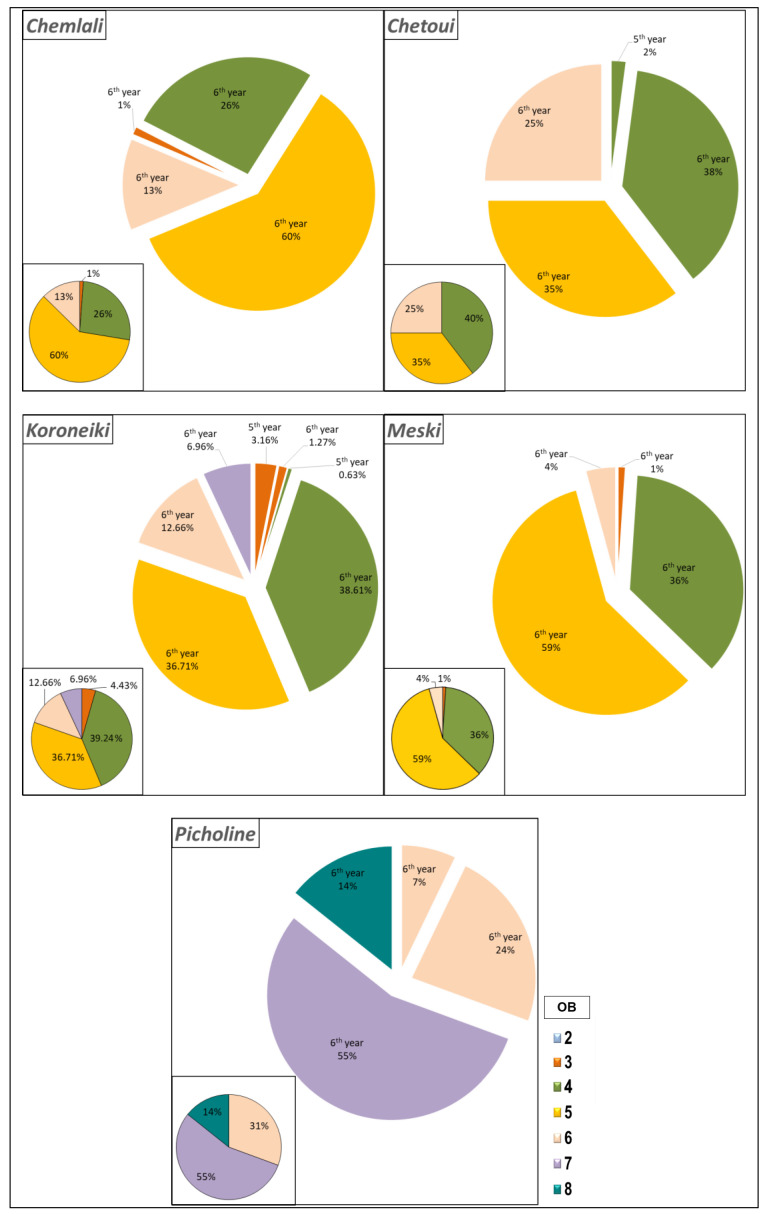
Distribution of inflorescences (Fl) (%) according to the botanical order (OB) per architectural unit of the cultivars *Chemlali*, *Chetoui*, *Meski*, *Koroneiki*, and *Picholine*.

According to the architectural study, 38.61% and 40% of the total number of inflorescences developed by the cultivars *Koroneiki* and *Chetoui*, respectively, were located in the OB4, whereas 60% and 59% of the total number of inflorescences developed by the cultivars *Chemlali* and *Meski*, respectively, were located in the OB5 (Figure 5). The cultivar *Picholine* produced 55% of the inflorescences in the OB7 (Figure 5). Regardless of the botanical order, all the inflorescences produced by the cultivars *Meski*, *Chemlali*, and *Picholine* were in the OB7. The cultivars *Koroneiki* and *Chetoui* produced 96.21% and 98% of their inflorescences on the 6th year (2015) developed shoots, respectively (Figure 5).

#### 2.1.4. Characterisation of Flowering and Fruiting Sites

**Figure 6:**
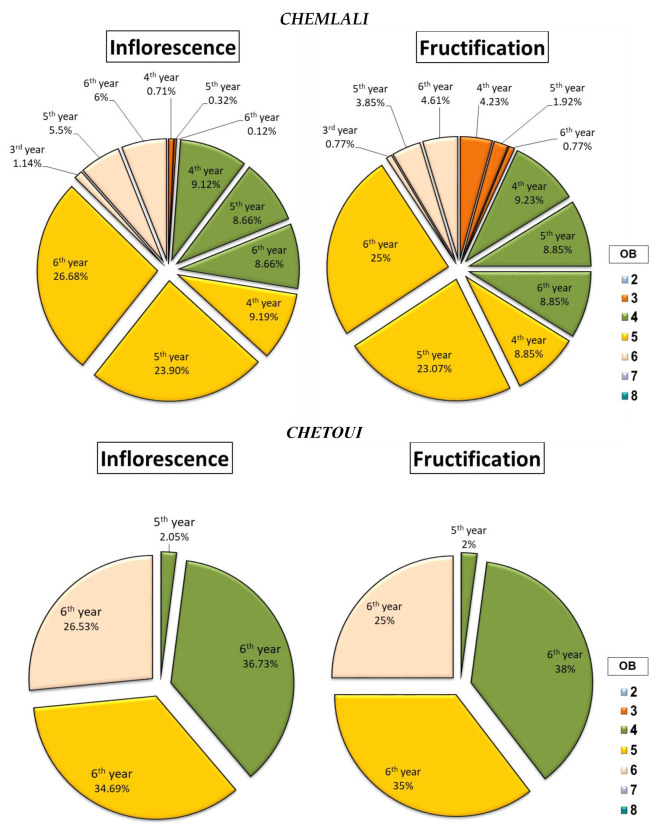
Distribution of inflorescences and fruits (%) according to the botanical order (OB) and year of development of the bearing shoot (year) per architectural unit of the cultivars *Chemlali* and *Chetoui*.

**Figure 7:**
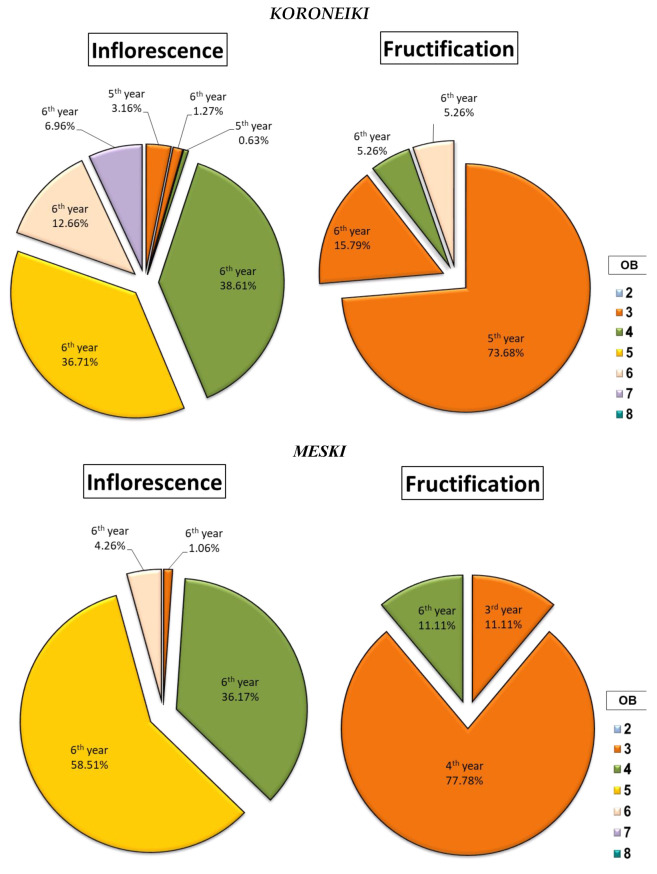
Distribution of inflorescences and fruits (%) according to the botanical order (OB) and year of development of the bearing shoot (year) per architectural unit of the *Koroneiki* and *Meski* cultivars.

**Figure 8:**
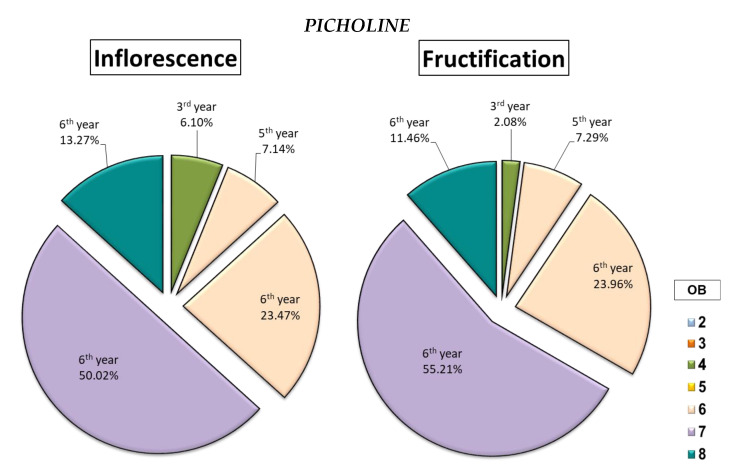
Distribution of inflorescences and fruits (%) by the botanical order (OB) and year of development of the bearing shoot (year) per architectural unit of the *Picholine* cultivar.

The study of the percentage of inflorescences and fruits gave a clearer idea about the ability of each cultivar to produce inflorescences and maintain them as fruits afterward. After studying the percentage of inflorescences and fruits according to both the botanical order and the year of development of the bearing branch, a clear difference was found between botanical orders for each cultivar (Figure 6, Figure 7 and Figure 8).

Indeed, the cultivars *Chemlali* and *Chetoui* produced the majority of their inflorescences as well as their fruits in the same botanical order (OB5 and OB4) respectively, with a percentage of 59.77% and 38.78% of the total inflorescences and 56.92% and 40% of the total fruits, respectively (Figure 6). The cultivar *Picholine* had the same tendency as the cultivars *Chetoui* and *Chemlali*, with 50.02% of the total inflorescences and 55.21% of the total fruits produced by the architectural units of the OB7 (Figure 8).

The *Meski* and *Koroneiki* cultivars behaved very differently. They produced the majority of their inflorescences and fruits in different botanical orders. The first cultivar (*Meski*) developed 58.51% of its inflorescences in the OB5 and 88.89% of its fruits in the OB3 (Figure 7), while the second cultivar (*Koroneiki*) developed 38.61% of its inflorescences in the OB4 and 89.47% of its fruits in the OB3 (Figure 7).

The data obtained from the architectural study showed a significant difference between the studied cultivars. Indeed, the cultivars *Meski* and *Koroneiki* produced between 77.78% and 73.68% of their fruits on the shoots of the 4th year (2013) and the 5th year (2014), respectively (Figure 7). The cultivars *Chemlali*, *Picholine*, and *Chetoui* produced 39.23% (Figure 6), 90.63% (Figure 8), and 98% (Figure 6) of their fruits on the 6th year (2015) twigs, respectively.

### 2.2. Biometric Characterisation of Long Shoots

#### 2.2.1. Variation of Shoot Length

The shoot length according to the botanical order showed a significant difference between the studied cultivars. The mean values of the annual shoot length measured at the level of the olive shoots in different botanical orders are grouped in Table 1, Table 2, Table 3, Table 4 and Table 5. The mean values of the total length of the shoots varied from 22.22 cm to 33.52 cm for the cultivars *Chetoui* (Table 3) and *Koroneiki* (Table 5), respectively. The lowest values of the total shoot length were recorded in the OB5 for the cultivars *Chemlali* (19.5 cm) (Table 1) and *Koroneiki* (19 cm) (Table 5) and in the OB6 for the cultivars *Chetoui* (6.25 cm) (Table 3) and *Picholine* (18.5 cm) (Table 4). The highest values of the total shoot length were recorded in the OB2 for the majority of the studied cultivars: *Chemlali* (72 cm) (Table 1), *Chetoui* (80 cm) (Table 3), *Meski* (78 cm) (Table 2), and *Koroneiki* (100 cm) (Table 5).

#### 2.2.2. Variation in the Number of Internodes

According to the botanical order, the number of internodes showed a significant difference between the studied cultivars. The average numbers of the internodes constructed by annual growths for different varieties and according to the botanical order are shown in Table 1, Table 2, Table 3, Table 4 and Table 5; the averages varied from 15 to 21 for the cultivars *Chemlali* and *Meski*, respectively. It is noteworthy that the length of shoots and the number of internodes decreased as the botanical order increased. On the other hand, it is noteworthy that the lowest number of internodes was in the OB6 for the cultivars *Chetoui* (11 E.N.) (Table 3) and *Picholine* (16 E.N.) (Table 4) and in the OB3, OB4, and OB5 for the cultivars *Chemlali* (14 E.N.) (Table 1), *Meski* (19 E.N.) (Table 2), and *Koroneiki* (13 E.N.), respectively. The highest number of internodes was in the OB2 for the cultivars *Chemlali* (41 E.N.) (Table 1), *Chetoui* (55 E.N.) (Table 3), *Meski* (50 E.N.) (Table 2), and *Koroneiki* (46 E.N.) (Table 5).

#### 2.2.3. Variation in Perennial Shoot Dimensions: Internode Length, Apex Diameter, and Basal Vigour

The average values of the parameters measured on olive shoots according to the botanical orders are grouped in Table 1, Table 2, Table 3, Table 4 and Table 5. The average internode length varied significantly between the cultivars, considering the botanical order. However, the average internode length was the highest for the cultivar *Koroneiki* (2.17 cm) in the OB2 (Table 5), while it was the lowest for the cultivar *Chetoui* (0.54 cm) in the OB6 (Table 3). Remarkably, the shortest internodes of the cultivars *Chemlali* (1.4 cm) (Table 1), *Meski* (1.03 cm) (Table 2), and *Koroneiki* (1.49 cm) (Table 5) were located in the OB5. The longest internodes were located in the OB2 of the cultivars *Chemlali* (1.76 cm) (Table 1), *Chetoui* (1.45 cm) (Table 3), *Meski* (1.56 cm) (Table 2), and *Koroneiki* (2.17 cm) (Table 5). The average basal shoot diameters for the studied cultivars are shown in Table 1, Table 2, Table 3, Table 4 and Table 5. For all the studied cultivars, it is clear that shoot vigour decreased with increasing botanical order. However, the lowest mean basal diameter was found either in the OB5 of the cultivars *Chemlali* (2.65 mm) (Table 1) and *Meski* (3.8 mm) (Table 2) or in the OB4, OB6, and OB7 of the cultivars *Koroneiki* (0.68 mm) (Table 5), *Chetoui* (1.66 mm) (Table 3), and *Picholine* (3.53 mm) (Table 4), respectively. The highest average basal diameters were marked in the OB2 of the cultivars *Chemlali* (20.6 mm) (Table 1), *Chetoui* (17.66 mm) (Table 3), *Meski* (18.5 mm) (Table 2), and *Koroneiki* (24 mm) (Table 5).

The mean shoot tip diameters of the studied cultivars are shown in Table 1, Table 2, Table 3, Table 4 and Table 5. Shoot tip diameters decreased with increasing botanical order, with a significant difference in their spatial location between the cultivars. The lowest average diameters at the tips were found either in the OB5 of the cultivars *Chemlali* (0.98 mm) (Table 1) and *Meski* (0.9 mm) (Table 2) or in the OB6 for the cultivars *Chetoui* (0.86 mm) (Table 4) and *Koroneiki* (1 mm) (Table 5). The highest averages were found either in the OB3 of the cultivars *Chemlali* (2.9 mm) (Table 1), *Chetoui* (1.55 mm) (Table 3), and *Meski* (1.93 mm) (Table 2) or in the OB5 of the cultivars *Picholine* (1.56 mm) (Table 4) and *Koroneiki* (5.97 mm) (Table 5).

### 2.3. Analysis of the Geometric Architectural Variability

**Figure 9:**
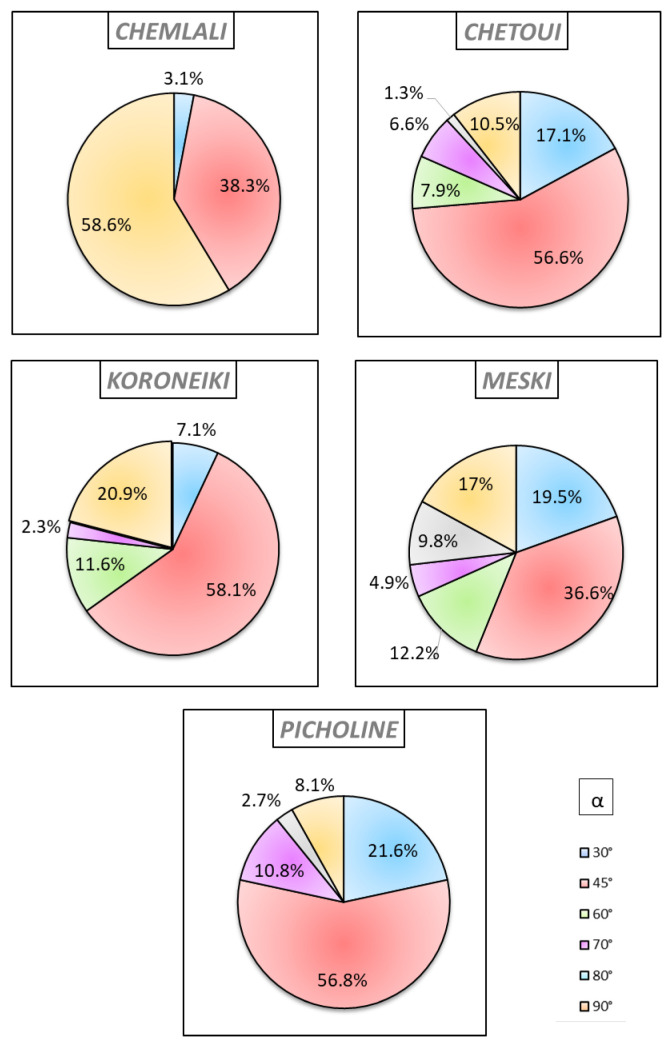
Variation of the branching insertion angle (α) (%) according to the botanical order (OB) per architectural unit of the cultivars *Chemlali*, *Chetoui*, *Koroneiki*, *Meski*, and *Picholine*.

**Figure 10:**
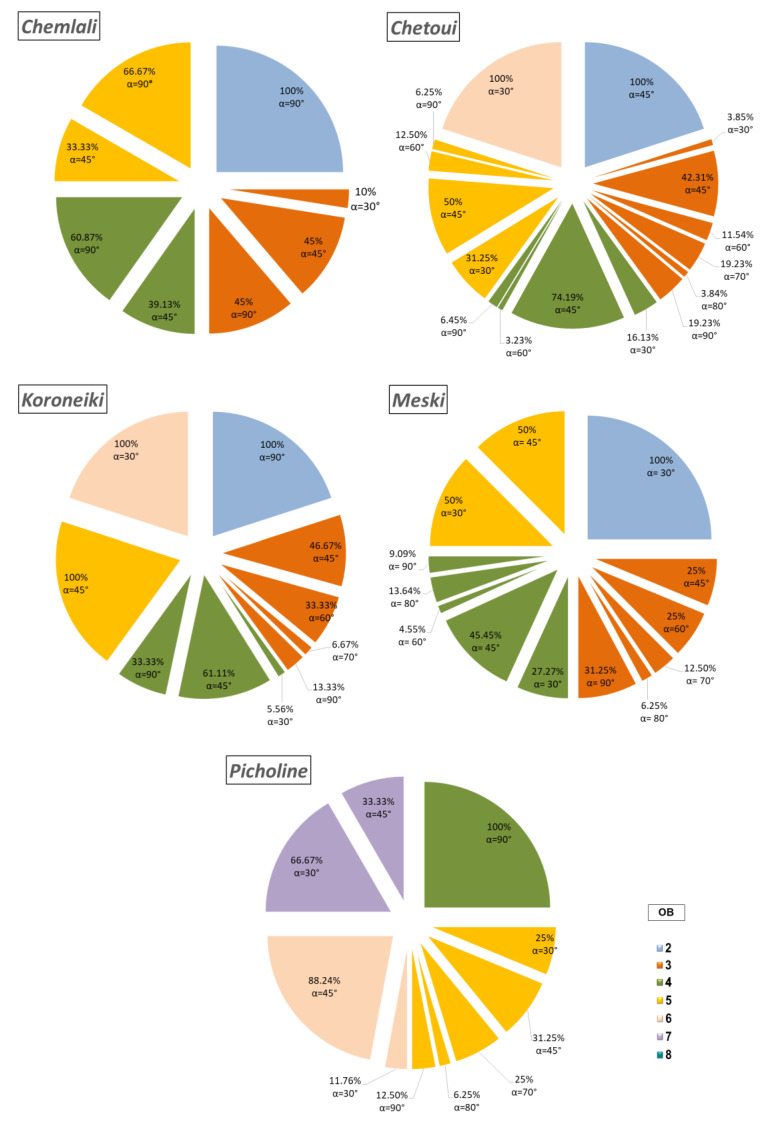
Variation of the branching insertion angle (α) (%) according to the botanical order (OB) per architectural unit of the *Chemlali*, *Chetoui*, *Meski*, *Koroneiki*, and *Picholine* cultivars.

The average insertion angle of the shoot with its supporting shoot showed a significant difference between the cultivars (Figure 9). In fact, this angle varied from 30° to 90°. For all the studied cultivars, the angle that the shoot made with its support shoot depended strongly on the botanical order (Figure 10). It is noteworthy that all shoots of the OB2 of the cultivar *Meski* and all shoots of the OB6 of the cultivars *Koroneiki* and *Chetoui* made an angle of 30° with their supporting branches (Figure 10). All shoots of the OB5 and the OB2 of *Koroneiki* and *Chetoui* cultivars, respectively, formed angles of 45° with their supporting shoots (Figure 10). All the OB2 stems of the cultivars *Chemlali* and *Koroneiki* and all the OB4 stems of the *Picholine* cultivar formed angles of 90° with their supporting stems (Figure 10). The cultivars *Chetoui* and *Picholine* developed 17.1% and 21.6%, respectively, of their shoots, with an insertion angle of 30° (Figure 9), of which 38.47% and 50%, respectively, were located in the OB5 of each cultivar (Figure S1). The cultivars *Chemlali*, *Meski*, and *Koroneiki* had percentages of 3.1%, 19.5%, and 7.1%, respectively (Figure 9), of the shoots creating angles of insertion of 30°, of which 100%, 87.5%, and 66.67% were located in OB3, OB4, and OB6, respectively (Figure S1). It is noteworthy that 38.3% (*Chemlali*), 56.6% (*Chetoui*), 36.6% (*Meski*), and 58.1% (*Koroneiki*) of the total number of shoots with angles of insertion were shoots with angles of insertion of 45° (Figure 9), of which 52.9% (*Chemlali*), 53.5% (*Chetoui*), 66.7% (*Meski*), and 44% (*Koroneiki*) were located in the OB4 of each cultivar (Figure S2). For the cultivars *Chetoui*, *Meski*, and *Koroneiki*, 7.9%, 12.20%, and 11.6%, respectively, of the shoots had branching angles of 60° (Figure 9), of which 50% (*Chetoui*), 80% (*Meski*), and 100% (*Koroneiki*) were found in the OB3 (Figure S3). Only 6.6% (*Chetoui*), 4.9% (*Meski*), and 2.3% (*Koroneiki*) of the total number of shoots developed an angle of 70° (Figure 9) and were all located in the OB3 of each cultivar (Figure S4). On the other hand, 1.3% (*Chetoui*), 2.7% (*Picholine*), and 9.8% (*Meski*) of the total number of shoots created insertion angles of 80° (Figure 9), of which 100% (*Chetoui*), 100% (*Picholine*), and 75% (*Meski*) were located in the OB3, OB3, and OB4, respectively (Figure S5). The largest possible angle that the shoots formed with their supports was 90° (Figure 9). In fact, 58.6% (*Chemlali*), 10.5% (*Chetoui*), 8.1% (*Picholine*), 17% (*Meski*), and 20.9% (*Koroneiki*) of the total number of shoots with insertion angles were branched creating insertion angles of 90° (Figure 9), of which 53.85% (*Chemlali*), 62.5% (*Chetoui*), 66.67% (*Picholine*), 71.43% (*Meski*), and 55.65% (*Koroneiki*) were located in the OB4, OB3, OB5, OB3 and OB5, respectively (Figure S6).

The distribution of the shoots according to their insertion angles as well as the year of development of their supporting shoots (Figures S1–S6), it was noticeable that all the shoots of the 3rd year (2011) for the cultivars *Koroneiki*, *Chemlali*, and *Meski* formed angles of 90° with their supporting shoots (Figure S6). For the cultivar *Koroneiki*, 100% of shoots made branching angles of either 60° (Figure S3) or 70° (Figure S4) with their supporting branches are shoots of the 4th year (2013), while for the cultivars *Chemlali* and *Picholine*, 100% of the shoots creating insertion angles of 30° (Figure S1) and 80° (Figure S5), respectively are shoots of the 4th year (2013). All the shoots of the cultivar *Meski* developed in the 6th year (2015) made angles of insertion of 30° (Figure S1) with their supporting branches. All the shoots of the cultivar *Picholine* developed in the 5th year (2014) made angles of insertion of 45° with their supporting shoots (Figure S2).

## 3. Discussion

This architectural study revealed a complex interaction between the topology of tree units (resulting from growth and branching processes) and their geometry, including shapes, spatial orientation of branches, and position of inflorescences and fruits within the architectural unit. This was possible thanks to the database developed based on a multistage coding of the architectural representation (topological coding plus geometric measurements).

### 3.1. Canopy Architecture Variability

The architectural parameters of the shoots (shoot length, internode length, etc.) did not seem to differ significantly between the olive cultivars studied when assessed from a global perspective (i.e., the overall average of the entire architectural unit). This finding is consistent with the results reported by Catania et al. [22] and Dhiab et al. [23], although it is important to contextualise this agreement. Specifically, Catania et al. [22] focused on canopy assessment using UAV multispectral imagery, which may involve different methods and environmental conditions, such as varying light exposure and terrain. Similarly, Dhiab et al. [23] studied olive trees under super high-density cropping systems, which could influence the expression of architectural traits. However, both studies assessed total canopy parameters such as shoot length and internode length, making their results comparable to ours in terms of the overall architectural structure. Despite potential differences in methodology and cropping systems, the consistency in the lack of significant differences between cultivars highlights the robustness of these architectural traits under different conditions and supports the idea that these parameters can be reliably used for comparative analyses in olive cultivation. However, it is necessary to deepen this general and global research towards the detailed study of these quantitative architectural parameters according to their distribution on the botanical orders, as the difference between cultivars became significant and clear as mentioned by Rosati et al. [13] who showed that each olive tree develops its shape through a specific growth pattern or “architectural model”, which represents its basic growth strategy. Analysis of a plant’s architecture is important for understanding its growth, branching pattern, and productivity, as well as for developing cultivation models. Branching by botanical order was found to be a relevant descriptor of architectural variability in olive, as previously shown for the 3rd botanical order in apple by Belhassine et al. [24]. Our study showed that the cultivars *Chemlali* and *Meski* had most of their shoots in the OB5, while the cultivars *Koroneiki* and *Chetoui* had most of their shoots in the OB4. These results contradict those obtained by Ahmad et al. [25], who showed that each olive cultivar had most of its shoots in a specific botanical order. The density of branches, as well as their position on the branches, varies greatly between cultivars, so each olive cultivar may be characterised by a specific branching mode, as suggested by Ahmad et al. [25] as well as by Rosati et al. [13], who confirmed that endogenous (i.e., genetic) factors vary between the cultivars of a species and influence the architecture of the plant. Furthermore, this study showed that the basal diameter is correlated to the node number of the shoot, while it is inversely correlated to branching frequency. These relationships differed from one cultivar to another. As a result, the cultivars *Chemlali* and *Koroneiki* have the thinnest shoots. In apple [26], the most branched trees have the lowest vigour and shoot dominance, while the least branched cultivars have the highest vigour and significant apical dominance.

### 3.2. Fruiting Characteristics

The olive cultivars showed similar branching characteristics but insufficient fruiting. Only when the architectural and fruiting characteristics are combined, it is possible to separate cultivars into groups, as proposed by Rosati et al. [13] to separate *Arbequina* and *Arbosana* from all other cultivars, and prove that both characteristics (canopy architecture and fruiting characteristics) are necessary to obtain high yields in small canopies and, therefore, suitability for SHD systems. The number of short internodes was characteristic of the *Koroneiki* cultivar, which may be a means of increasing canopy density and the number of potential fruiting sites per unit canopy volume. The intensity of inflorescences and fruits and their positions in the architectural unit are architectural traits associated with cultivar preference [27]. As a result of our study, it was noteworthy that the cultivars *Chemlali*, *Chetoui*, and *Picholine* produced the majority of their inflorescences and fruits on the same botanical orders, while the rest of the studied cultivars (*Meski* and *Koroneiki*) did so on different botanical orders (usually two successive orders). The results of this architectural study proved that the number of inflorescences and the number of fruits per architectural unit are inversely proportional to the basal diameter of the bearing shoots for the cultivars *Chemlali* and *Koroneiki*. This means that these two cultivars have a high number of fruits and thin basal branches. This suggests that these cultivars, according to the hypothesis of Dhiab et al. [23], with high branching and small basal diameters, have important architectural characteristics to increase yield efficiency and influence the suitability of cultivars for hyper-intensive orchards.

### 3.3. Plant Vigour and Geometric Architectural Variability

From this study on branching growth and reproductive characteristics, it should be noted that the latter two are closely related to the morphology of the bearing axes and their spatial positions, as revealed by Catania et al. [22] and Rosati et al. [13]. Indeed, according to Rosati et al. [26], high values of branching frequency imply, on the one hand, a greater capacity to fill the canopy volume with potential fruiting sites and, on the other hand, a reduction in the number of fruiting site structures. In other species, greater branching with finer structures leads to greater flowering and fruiting because less vigorous shoots fill the canopy volume, leading to more productive sites [26]. Nevertheless, these results suggest an early physiological aging of the canopy due to excessive shading, as shown in the architectural study of the cultivars *Koroneiki*, *Arbequina*, *Arbosana*, and FS17 [8]. The angle of shoot insertion on the bearing shoots does not vary much between cultivars *Meski*, *Chetoui*, and *Koroneiki*, suggesting that these cultivars are suitable for planting in the same orchard. This homogeneity in spatial development will facilitate the mechanisation of tree maintenance and fruit harvesting. Similar suggestions have been made by Rosati et al. [26] for the cultivars *Arbequina* and *Arbosana*, recommended for SHD (super-high-density) orchards.

The monitoring of the angle of insertion of the shoots on their bearing shoots showed that this parameter does not vary much between the cultivars *Chetoui* and *Koroneiki*, which suggests that these cultivars are suitable to be planted in the same orchard, with possibilities of mechanisation of maintenance and harvesting.

This study can be a first outline, on the one hand, to elaborate basic guidelines for modern management (optimal distances between trees, necessary contribution in fertigation and fertilisation), which will bring new satisfactory and sustainable answers to the good management of olive orchards in Tunisia. On the other hand, the approach adopted in the present study, detailing the spatial and temporal distribution of branching, flowering, and fruiting, will greatly help biologists to determine the right time in the development of the plant when morphological changes are sufficiently important to be distinguished by statistical studies. This will provide objective criteria for the design of sampling procedures. For physiologists, such a study will pave the way to predict the relationships between architectural differences and physiological mechanisms (photosynthesis, solar radiation interception, and fruit yield). The results concerning the spatiotemporal concentration of branching, inflorescence, and fruiting will help entomologists to ensure a better understanding of the architecture of each olive cultivar, thus having a more precise idea of the areas at risk of disease or attack by pathogens or pests. Moreover, this study is well-adapted to the optimisation programmes of the architectural modelling software of trees, and especially of olive trees. From a practical agronomic point of view, the results of this study provide valuable insights for optimising the architectural control of different olive cultivars, which is crucial for improving tree growth management and increasing overall orchard productivity.

Some limitations of this study should be highlighted, including the relatively small sample size and the lack of associated physiological data (photosynthesis, light interception, etc.) that could complement the interpretation of the results. These elements should be further investigated in future work in order to validate and generalise the trends observed.

## 4. Materials and Methods

### 4.1. Plant Material and Growth Conditions

The experimental design was based on five olive cultivars (*Olea europaea* L.); two introduced cultivars (*Koroneiki*, *Picholine*) and three local cultivars (*Chemlali*, *Chetoui*, *Meski*) at the Taous research station, about 40 km from Sfax, in central Tunisia (34.93° N 10.61° E; 120 m above sea level), with a semi-arid climate [28]. The mean temperature was 23.2 °C, precipitation was about 203 mm yr^−1^, and ETO was ca. 1400 mm yr^−1^ [28]. The planting density was 204 trees ha^−1^ [28]. The soil consisted of 76% sand, 14% silt, and 10% clay. The trees were trained in an open vase and grown under rainfed conditions [28]. Three architectural units [9] were chosen, with three trees of each variety aged 15 years.

### 4.2. Measured Parameters

The spatiotemporal evolution of branching was monitored by assessing the branching capacity within each growth unit. Subsequently, for each architectural unit, the branching density (the number of branches developed along a given axis per unit of length of that axis) [28] and the extent of branching (illustrated by the average percentage of shoots developed at the level of the units studied) [9] were derived. Table 6 is an illustrative example of the topographical data coding method used during the study.

### 4.3. Architectural Studies

**Figure 11:**
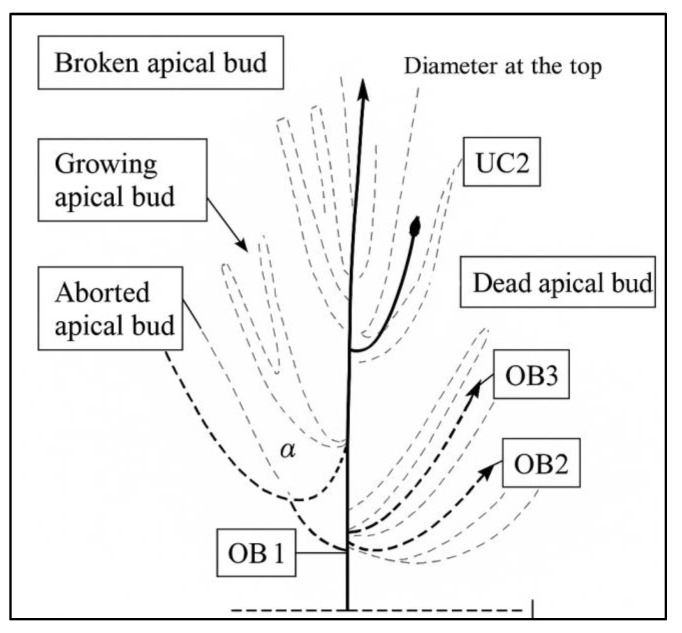
Primordial architectural description of the architectural unit. CU: growth unit; OB: botanical order; α: angle of insertion of the shoot on the bearing shoot.

Description of the different levels of organisation: The description of three architectural units [9] per tree at a rate of three trees per variety was carried out with very simple equipment: a tape measure, a digital compass, and a computer. The study was conducted on the growth units [9] selected for each tree [13,29], with observations focusing on shoot development over time. The influence of time was determined by comparing the ability of successive growth units [9] to produce branches, which is informed by the type of branching as well as its nature (short shoot, long shoot), then concerning space (by analysing the ability to produce shoots within each growth unit), and then deducing for each growth unit the intensity of branching (number of shoots formed) and the location of branching (the distribution of these shoots along the unit studied) (Figure 11). The acquisition of data is the starting point for the constitution of the database. The shoots of the olive tree were described from the inside to the outside of the canopy. A topographic coding of these data was then performed [16,29]. These data were organised in an Excel^®^ file according to the MTG coding defined by Annabi [9] and Gaaliche et al. [29], whose structure includes two parts. The first part concerns the position of the architectural unit on the tree, and the second part concerns the different parameters measured for each botanical order (refers to the hierarchical level of branching within a plant’s shoot system [8,9,14]).

### 4.4. Analysis of Topographic Architectural Variability

The main architectural parameters studied are as follows: vegetative growth (the production and elongation of new shoots and the thickening of stems [9]) in each botanical order, branching (the growth of one or more new morphological units similar to the generative unit [9]), morphological differentiation of axes (refers to how branches specialise into vegetative or reproductive functions, shaping the overall tree architecture [13,30]), and both apical (at the tip) and lateral (along the sides) positions of reproductive structures (whether they influence flowering patterns, fruit distribution, and orchard management efficiency, especially in high-density systems). These traits are crucial for selecting cultivars adapted to mechanisation and sustainable production [13,30]. This determines the extent of branching, illustrated by the average percentage of shoots developed at the levels of the units studied [9], the branching density, which is the position of shoots by botanical order at the scale of the supporting axis [9], the characteristics of the supporting shoots (supporting shoot: shoot of a lower order (OBn-1) than the studied shoot (OBn); it is generally a more vigorous shoot [9]), and the distribution of flowering and fruiting on the different botanical orders.

### 4.5. Annual Growth Analysis

This analysis was performed at the twig scale and allows for determining shoot length (annual growth), number of internodes, and variation in multi-year shoot dimensions (length per internode, diameter at the top, and diameter at the base) [9].

### 4.6. Analysis of Geometric Architectural Variability

This analysis was performed at the spatial scale by taking into account the neighbourhood effect between shoots and their spatial distributions [13]. The two main parameters determined were the variation of the insertion angle and the variation of the state at the tip.

### 4.7. Statistical Analysis

Quantitative data were expressed as the means ± standard errors (SE), calculated on the basis of at least three replicates (n ≥ 3) per sample. Statistical analyses were performed using SPSS 20.5 software (SPSS Inc., Chicago, IL, USA). One-way or two-way fixed analysis of variance (ANOVA) was performed, followed by Duncan’s multiple range test to compare the means. All results are presented as the means ± standard deviations of at least three measurements. Significant differences between the treatments were determined at a significance level of *p* < 0.05, with statistical significance assessed using Duncan’s test.

## 5. Conclusions

The study of qualitative and quantitative architectural traits provides valuable insights into the factors influencing the growth and productivity of olive trees. Our analysis revealed significant differences between olive cultivars in architectural traits such as branch insertion angles, shoot lengths, and inflorescence distribution. These differences are primarily due to genetic factors, but environmental conditions also play a key role in shaping the final architectural structure.

While the direct application of these findings to orchard management practices is still being explored, we suggest that a deeper understanding of architectural traits can inform more targeted management approaches. For example, knowing the typical angle of branch insertion of a cultivar may influence decisions on pruning methods and tree spacing in high-density orchards, potentially optimising light penetration and improving overall tree vigour. Similarly, the proportion of long shoots could inform pruning strategies to improve canopy uniformity, which may ultimately improve fruit production.

Our results also highlight the importance of considering cultivar compatibility when planning multi-cultivar orchards. Understanding architectural coherence between cultivars can help ensure uniform growth patterns, reducing management complexity and improving mechanisation efficiency for tasks such as pruning and harvesting.

In addition, the results highlight the need for more accurate modelling of tree architecture, particularly for olive trees, to support the development of precision farming techniques. This includes refining models that can predict canopy development and guide management decisions, such as irrigation and fertilisation, based on the specific architectural needs of each cultivar. Finally, this study paves the way for future molecular studies aimed at identifying genetic markers associated with architectural traits, which could provide further insights into how these traits are inherited and how they can be optimised in breeding programmes.

## Figures and Tables

**Table 1:** Variation of quantitative parameters (lg: average total length of the branch; Nbre E.N.: average number of the internodes; Diam B: average basal diameter; Diam S: average diameter of the apex; L.E.N.: average length of the internodes) as a function of the botanical order (OB) and year of development of the bearing shoots (year) in the olive cultivar *Chemlali*.

*Chemlali*		Quantitative Parameters				
OB	Year	Lg	Nbre E.N.	Diam B	Diam S	L.E.N.
2	3	72 ± 0.00 ^a^	41 ± 0.0 ^a^	20.6 ± 0.0 ^a^	2 ± 0.0 ^c^	1.76 ± 0.0 ^a^
3	4	21.2 ± 9.17 ^c^	13.45 ± 1.4 ^d^	5.17 ± 2 ^c^	2.9 ± 1.58 ^b^	1.71 ± 0.7 ^b^
4	4	25.75 ± 6.1 ^b^	14.94 ± 8.0 ^b^	7.58 ± 1 ^b^	14.7 ± 5.2 ^a^	1.65 ± 0.4 ^c^
5	4	19.5 ± 3.11 ^d^	14.25 ± 2 ^c^	2.65 ± 1 ^d^	0.98 ± 0.05 ^d^	1.4 ± 0.08 ^d^
Average		24.84 ± 8.04	15.16 ± 1.10	6.54 ± 0.78	8.53 ± 3.64	1.64 ± 0.49

Different letters (^a^, ^b^, ^c^ and ^d^) indicate significant differences between different botanical orders (OB) (*p* ≤ 0.05) for each parameter.

**Table 2:** Variation of quantitative parameters (lg: average total length of the branch; Nbre E.N.: average number of the internodes; Diam B: average basal diameter; Diam S: average diameter of the apex; L.E.N.: average length of the internodes) as a function of the botanical order (OB) and year of development of the bearing shoots (year) in the olive cultivar *Meski*.

*Meski*		Quantitative Parameters				
OB	Year	Lg	Nbre E.N.	Diam B	Diam S	L.E.N.
2	4	78 ± 0.0 ^a^	50 ± 0.0 ^a^	18.5 ± 0.0 ^a^	1.5 ± 0.0 ^c^	1.56 ± 0.0 ^a^
3		24.31 ± 3.71 ^c^	22.13 ± 1.23 ^c^	5.41 ± 1.81 ^c^	1.93 ± 0.13 ^c^	1.11 ± 0.24 ^c^
3	3	25 ± 0.0	22 ± 0.0	5.8 ± 0.0	3.9 ± 0.0	1.14 ± 0.0
3	4	24.27 ± 4.18	22.13 ± 12.74	5.38 ± 1.87	1.79 ± 0.29	1.11 ± 0.25
4		21.14 ± 9.40 ^d^	19.14 ± 8.34 ^d^	5.89 ± 0.57 ^b^	1.52 ± 0.74 ^b^	1.17 ± 0.32 ^b^
4	4	20.47 ± 9.10	18.71 ± 8.30	5.94 ± 0.84	1.55 ± 0.74	1.17 ± 0.33
4	6	35 ± 0.0	28 ± 0.0	4.8 ± 0.0	0.9 ± 0.0	1.25 ± 0.0
5	4	24.5 ± 7.78 ^b^	23.5 ± 4.95 ^b^	3.8 ± 0.71 ^d^	0.9 ± 0.0 ^d^	1.03 ± 0.11 ^d^
Average		23.93 ± 3.99	21.27 ± 1.08	5.90 ± 0.47	1.65 ± 1.02	1.15 ± 0.29

Different letters (^a^, ^b^, ^c^ and ^d^) indicate significant differences between different botanical orders (OB) (*p* ≤ 0.05) for each parameter.

**Table 3:** Variation of quantitative parameters (lg: average total length of the branch; Nbre E.N.: average number of the internodes; Diam B: average basal diameter; Diam S: average diameter of the apex; L.E.N.: average length of the internodes) as a function of the botanical order (OB) and year of development of the bearing shoots (year) in the olive cultivar *Chetoui*.

*Chetoui*		Quantitative Parameters				
OB	Year	Lg	Nbre E.N.	Diam B	Diam S	L.E.N.
2		80 ± 0.0 ^a^	55 ± 0.0 ^a^	17.6 ± 0.0 ^a^	1.5 ± 0.0 ^b^	1.45 ± 0.0 ^a^
2	3_4	80 ± 0.0	55 ± 0.0	17.6 ± 0.0	1.5 ± 0.0	1.45 ± 0.0
3		27.37 ± 6.96 ^b^	19.96 ± 1.13 ^b^	4.17 ± 1.65 ^b^	1.55 ± 0.94 ^a^	1.43 ± 0.54 ^b^
3	3	26.57 ± 9.6	14.86 ± 1.14	4.11 ± 2.27	1.46 ± 0.12	1.82 ± 0.52
3	4	28.28 ± 6.65	22.67 ± 1.07	4.28 ± 1.42	1.61 ± 0.89	1.24 ± 0.44
3	5	15 ± 0.0	7 ± 0.0	2.6 ± 0.0	1.2 ± 0.0	2.14 ± 0.00
4		20.85 ± 7.81 ^c^	17.81 ± 6.28 ^c^	3.4 ± 0.91 ^b^	1.29 ± 0.7 ^c^	1.22 ± 0.42 ^d^
4	3	21.61 ± 1.28	18.78 ± 7.03	3.61 ± 0.87	1.51 ± 0.90	1.15 ± 0.41
4	4	20.4 ± 6.28	17.6 ± 6.26	3.33 ± 0.97	1.22 ± 0.6	1.22 ± 0.38
4	5	22 ± 7.07	15.5 ± 4.95	3.2 ± 0.28	0.95 ± 0.07	1.57 ± 0.96
5		14.97 ± 8.11 ^d^	12.94 ± 3.87 ^d^	2.65 ± 0.56 ^b^	1.06 ± 0.49 ^d^	1.25 ± 0.78 ^c^
5	3	10 ± 3.61	13 ± 1.73	2.82 ± 0.59	0.93 ± 0.41	0.80 ± 0.38
5	4	16.12 ± 8.51	12.92 ± 4.27	2.61 ± 0.57	1.09 ± 0.52	1.35 ± 0.82
6	4	6.25 ± 1.06 ^e^	11.5 ± 2.12 ^e^	1.66 ± 0.04 ^c^	0.86 ± 0.0 ^e^	0.54 ± 0.01 ^e^
Average e		22.22 ± 4.34	17.84 ± 9.38	3.65 ± 2.08	1.32 ± 0.75	1.28 ± 0.56

Different letters (^a^, ^b^, ^c^, ^d^ and ^e^) indicate significant differences between different botanical orders (OB) (*p* ≤ 0.05) for each parameter.

**Table 4:** Variation of quantitative parameters (lg: average total length of the branch; Nbre E.N.: average number of the internodes; Diam B: average basal diameter; Diam S: average diameter of the apex; L.E.N.: average length of the internodes) as a function of the botanical order (OB) and year of development of the bearing shoots (year) in the olive cultivar *Picholine*.

*Picholine*		Quantitative Parameters				
OB	Year	Lg	Nbre E.N.	Diam B	Diam S	L.E.N.
4	3	39 ± 0.0 ^b^	32 ± 0.0 ^a^	14 ± 0.0 ^a^	1.4 ± 0.0 ^b^	1.22 ± 0.0 ^a^
5		43.38 ± 6.30 ^a^	20.25 ± 7.98 ^c^	4.31 ± 1.68 ^a^	1.56 ± 0.13 ^a^	1.95 ± 1.20 ^a^
5	3	73.75 ± 5.16	24 ± 6.16	4.35 ± 1.66	1.1 ± 0.20	2.99 ± 1.09
5	4	34.64 ± 2.56	19.45 ± 8.59	4.27 ± 1.85	1.77 ± 1.53	1.63 ± 0.61
5	5	18 ± 0.0	14 ± 0.0	4.6 ± 0.0	1 ± 0.0	1.29 ± 0.0
6		18.5 ± 1.03 ^d^	15.82 ± 5.10 ^d^	3.61 ± 0.94 ^a^	1.11 ± 0.3 ^c^	1.23 ± 0.40 ^a^
6	3	18.67 ± 8.50	19.66 ± 5.03	3.93 ± 0.40	1 ± 0.0	0.98 ± 0.05
6	4	20.25 ± 1.27	15.08 ± 5.21	3.65 ± 1.05	1.13 ± 0.36	1.31 ± 0.37
6	5	17 ± 1.13	14.5 ± 3.54	2.85 ± 0.35	1.1 ± 0.14	1.11 ± 0.51
7	4	29.33 ± 2.52 ^c^	22 ± 3.46 ^b^	3.53 ± 0.25 ^a^	1 ± 0.0 ^d^	1.34 ± 0.11 ^a^
Average		30.37 ± 6.97	18.67 ± 7.04	4.19 ± 2.11	1.3 ± 0.89	5.74 ± 0.22

Different letters (^a^, ^b^, ^c^ and ^d^) indicate significant differences between different botanical orders (OB) (*p* ≤ 0.05) for each parameter.

**Table 5:** Variation of quantitative parameters (lg: average total length of the branch; Nbre E.N.: average number of the internodes; Diam B: average basal diameter; Diam S: average diameter of the apex; L.E.N.: average length of the internodes) as a function of the botanical order (OB) and year of development of the bearing shoots (year) in the olive cultivar *Koroneiki*.

*Koroneiki*		Quantitative Parameters				
OB	Year	Lg	Nbre E.N.	Diam B	Diam S	L.E.N.
**2**	**3**	100 ± 0.0 ^a^	46 ± 0.0 ^a^	24 ± 0.0 ^a^	1.2 ± 0.0 ^c^	2.17 ± 0.0 ^a^
**3**	**4**	48.47 ± 5.62 ^b^	23.53 ± 11.5 ^b^	5.77± 2.58 ^c^	1.79 ± 1.42 ^b^	2.13 ± 0.76 ^a^
**4**		23.75 ± 9.31 ^d^	15.44 ± 7.06 ^d^	0.68 ± 0.0 ^e^	1.09 ± 0.32 ^d^	1.69 ± 0.74 ^a^
**4**	**4**	23.67 ± 8.78	16 ± 1.09	3.77 ± 0.71	1.32 ± 0.49	1.90 ± 1.08
**4**	**5**	23.79 ± 9.94	15.17 ± 4.69	3.18 ± 0.62	0.98 ± 0.05	1.58 ± 0.53
**5**		19 ± 8.66 ^e^	13.43 ± 7.59 ^e^	8.89 ± 1.46 ^b^	5.97 ± 1.32 ^a^	1.49 ± 0.54 ^a^
**5**	**4**	2 ± 0.00	2 ± 0.0	42 ± 0.0	36 ± 0.0	1 ± 0.0
**5**	**5**	21.83 ± 4.75	15.33 ± 6.22	3.37 ± 0.59	0.97 ± 0.08	1.58 ± 0.54
**6**	**5**	27 ± 4.24 ^c^	16 ± 0.0 ^c^	3.5 ± 0.0 ^d^	1 ± 0.0 ^e^	1.69 ± 0.27 ^a^
**Average**		33.52 ± 2.98	18.67 ± 1.04	5.60 ± 0.67	2.13 ± 0.53	1.82 ± 0.27

Different letters (^a^, ^b^, ^c^, ^d^ and ^e^) indicate significant differences between different botanical orders (OB) (*p* ≤ 0.05) for each parameter.

**Table 6:** Example of topographical data coding.

MAIN AXIS								ORDRE 2						ORDRE3						ORDRE				QUALITATIVE PARAMETERS			QUANTITATIVE PARAMETRES					
Exp	Var	n° Arbre	Treat	OB-T	UC1	O1-ON	OB- U	nd br B2	nd br S2	OB-T	UC2	O2-ON	OB- U	nd br B3	nd br S3	OB	UC3	O3-ON	OB- U	UC	Year	OB-T	OB-U	NAT	State of the Bud	Nb. Fruit	lg	Nbre EN	diam B	diam S	Ang/Insert	LEN
ettaoues	1	15	3.1	4	UC13	100,000	1													UC13	3	4	1	2	2	97	39	32	14	1.4	90	1.22
ettaoues	1	15	3.1	4	UC13	120,000	1	12	21	5	UC24	20,000	2							UC24	4	5	2	2	2		17	13	2.4	0.9	80	1.31
ettaoues	1	15	3.1	4	UC13	120,000	1	12	21	5	UC26	20,000	2							UC26	6	5	2	1	3							
ettaoues	1	15	3.1	4	UC13	120,000	1	13	20	5	UC24	20,000	2							UC24	4	5	2	2	1		38	16	7.4	5.7	70	2.38
ettaoues	1	15	3.1	4	UC13	123,000	1	13	20	5	UC24	23,000	2	2	15	6	UC35	3000	3	UC35	5	6	3	1	2							
ettaoues	1	15	3.1	4	UC13	123,000	1	13	20	5	UC24	23,000	2	2	15	6	UC36	3000	3	UC36	6	6	3	3		2						
ettaoues	1	15	3.1	4	UC13	123,000	1	13	20	5	UC24	23,000	2	5	12	6	UC35	3000	3	UC35	5	6	3	1	2							
ettaoues	1	15	3.1	4	UC13	123,000	1	13	20	5	UC24	23,000	2	5	12	6	UC36	3000	3	UC36	6	6	3	1	3							
ettaoues	1	15	3.1	4	UC13	123,000	1	13	20	5	UC24	23,000	2	13	4	6	UC34	3000	3	UC34	6	6	3	1	2							
ettaoues	1	15	3.1	4	UC13	123,000	1	13	20	5	UC24	23,000	2	13	4	6	UC36	3000	3	UC36	6	6	3	1	3							
ettaoues	1	15	3.1	4	UC13	123,000	1	13	20	5	UC24	23,000	2	16	1	6	UC34	3000	3	UC34	4	6	3	2	2		13	12	3.9	1	30	1.09
ettaoues	1	15	3.1	4	UC13	123,400	1	13	20	5	UC24	23,400	2	16	1	6	UC34	3400	3	UC44	4	7	4	2	2		27	20	3.3	1	30	1.35

Exp: experimental site; Var: variety (1: *Picholine*; 2: *Koroneiki*; 3: *Meski*; 4: *Chetoui*; 5: *Chemlali*); n° arbre: number of the tree in the field; Treat: treatment; OB-T: botanical order in the tree (1: 4th botanical order; 2: 5th botanical order; 3: 6th botanical order; 4: 7th botanical order; 5: 8th botanical order; 6: 9th botanical order; 7: 10th botanical order; 8: 11th botanical order); UCij: growth unit of order “i” developed in year “j”; Oi-ON: order i in succession with the orders inserted on order I; OB-U: botanical order at the level of the architectural unit; nd br Bi: number of the node from the base of the twig of order i; nd br Si: number of the node from the top of the twig of order i; Year: year of the development of the branching (1: 2009; 2: 2010; 3: 2011; 4: 2013; 5: 2014; 6: 2015; 7: 2016); Nat: type of branching (1: short shoot; 2: long shoot; 3: inflorescence; 4: fruit); State of the bud: State of the apical bud of the shoot (1: aborted; 2: growing; 3: broken; 4: dead; 5: pruned); Nb.Fruit: number of the fruit on the branch; lg: length of the branch; Nb.E.N: number of internodes; diam B: base diameter; diam S: apex diameter; ang/insert: angle of insertion of the branching in relation to the supporting shoot; L.E.N: average length of the internode.

## Data Availability

The datasets used in this study are available from the corresponding author upon reasonable request.

## Supplementary Materials

The following supporting information can be downloaded at: https://www.mdpi.com/article/10.3390/plants14111707/s1.
