# Supplementary material for: Architectural Analysis for Novel Olive Crop Management
Source: Plants (Basel). 2025 Jun 3;14(11):1707. doi: 10.3390/plants14111707 (PMC12157064; doi:10.3390/plants14111707)
Supplement: Supplementary file 1 [file plants-14-01707-s001.zip › plants-3570542-supplementary.pdf]

Supplementary Document

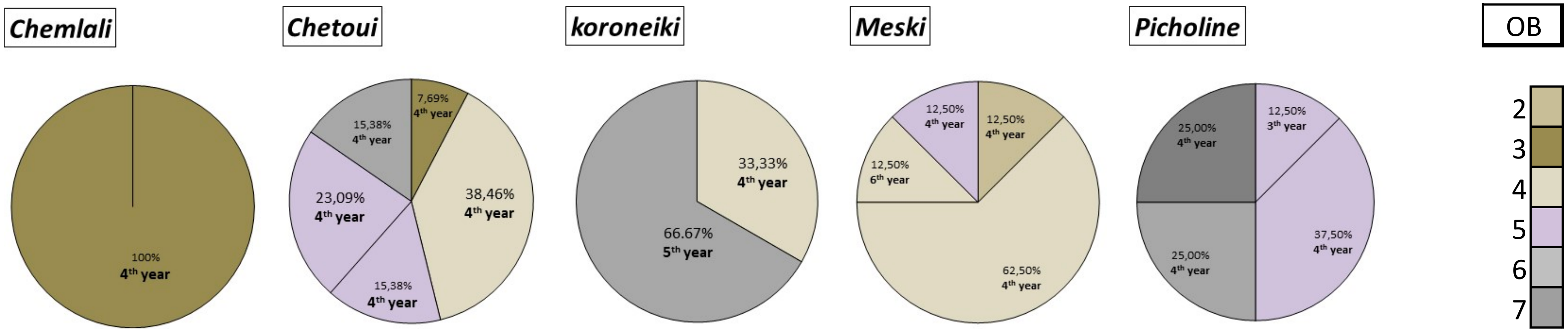

Figure S1. Distribution (in %) of branches with an insertion angle α= 30°, according to botanical order (OB) and year of development of the bearing branch (Year) by architectural unit of cultivars *Chemlali, Chetoui, Meski, Koroneiki* and *Picholine.*

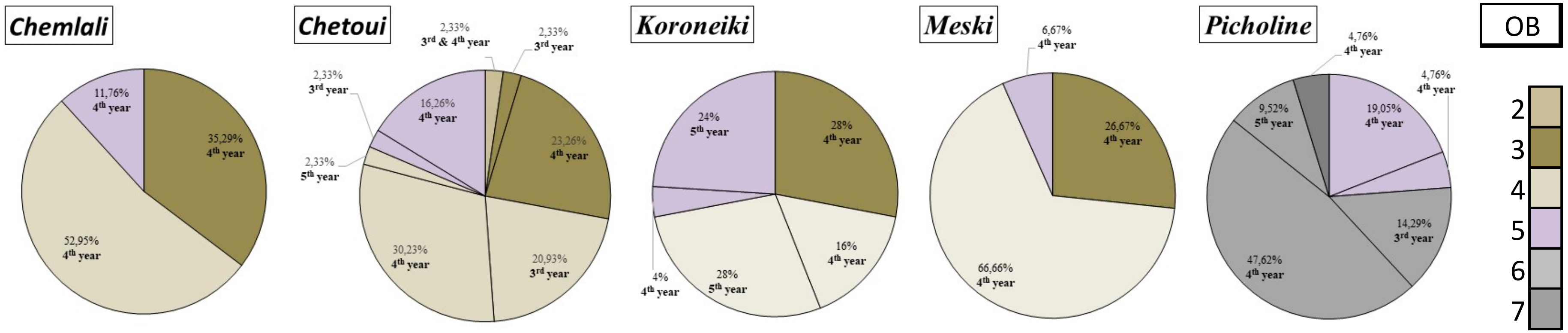

Figure S2. Distribution (in %) of branches with an insertion angle α= 45°, according to botanical order (OB) and year of development of the bearing branch (Year) by architectural unit of cultivars *Meski*, *Chemlali, Chetoui, Koroneiki*et*Picholine.*

Supplementary Document

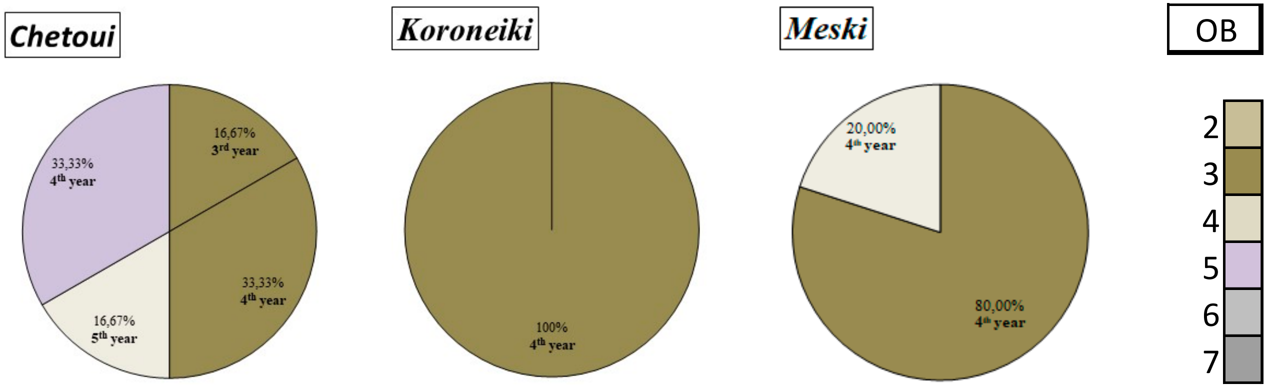

Figure S3. Distribution (in %) of branches with an insertion angle α= 60°, according to botanical order (OB) and year of development of the bearing branch (Year) by architectural unit of cultivars *Meski, Chetoui*and*Koroneiki.*

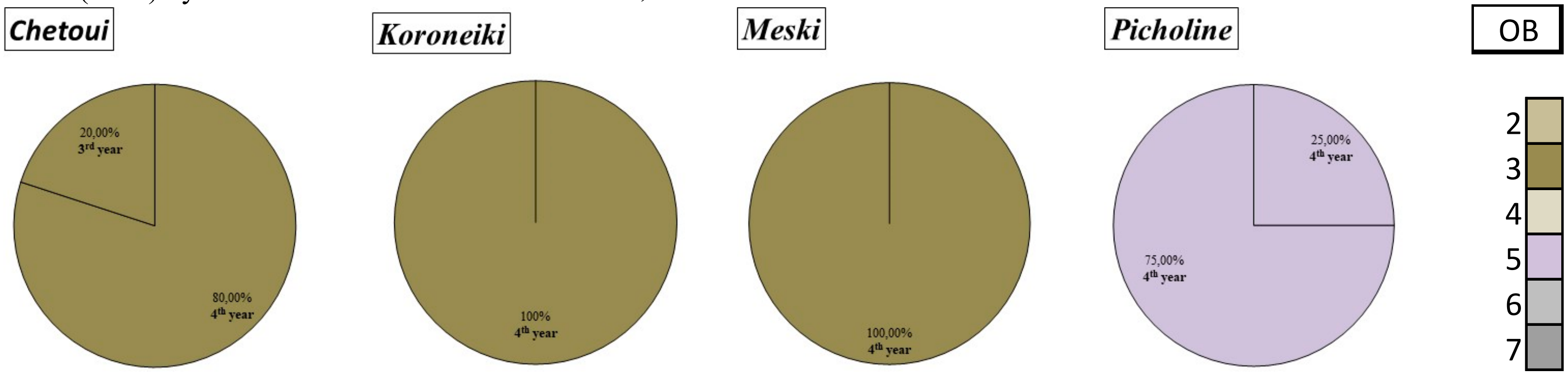

Figure S4. Distribution (in %) of branches with an insertion angle α= 70°, according to botanical order (OB) and year of development of the bearing branch (Year) by architectural unit of cultivars*Meski, Chetoui, Koroneiki* and *Picholine.*

Supplementary Document

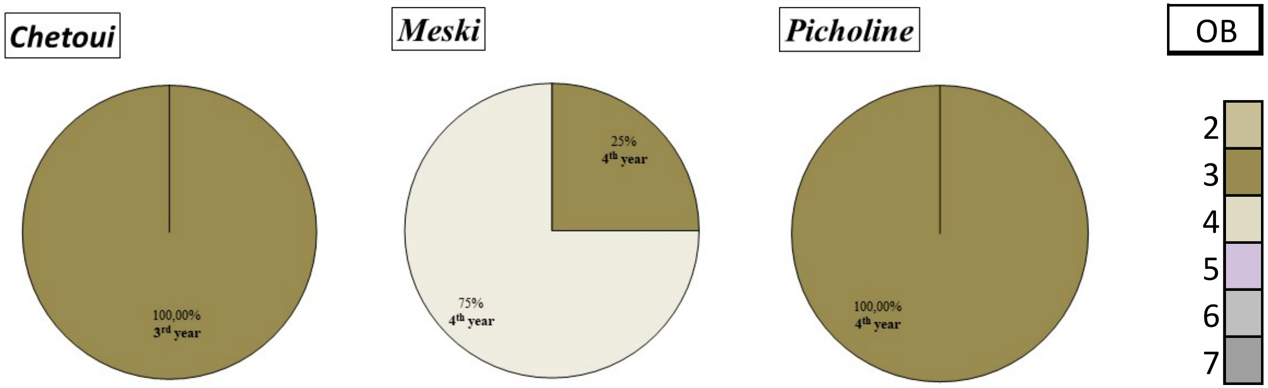

Figure S5. Distribution (in %) of branches with an insertion angle α= 80°, according to botanical order (OB) and year of development of the bearing branch (Year) by architectural unit of cultivars *Chetoui, Meski*and*Picholine.*

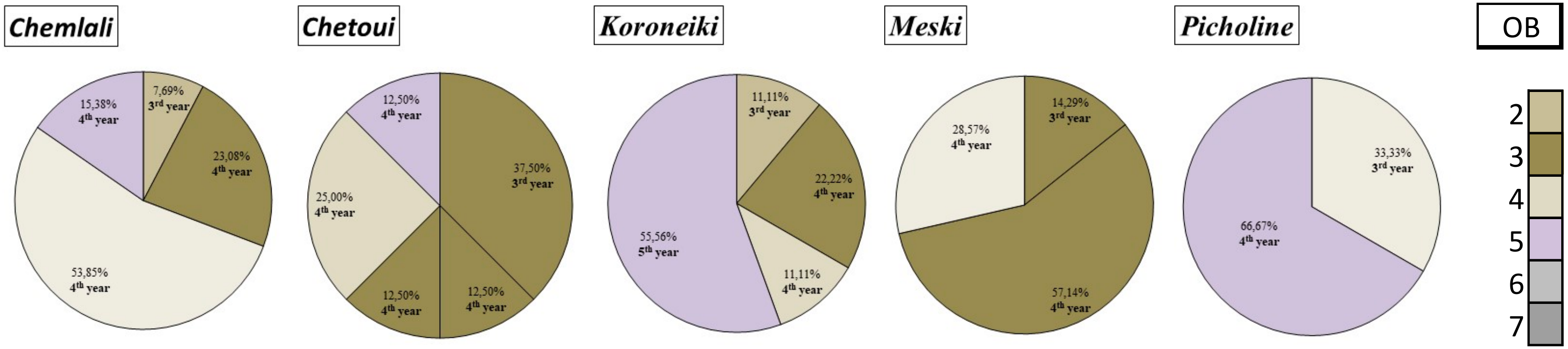

Figure S6. Distribution (in %) of branches with an insertion angle α= 90°, according to botanical order (OB) and year of development of the bearing branch (Year) by architectural unit of cultivars *Meski, Chemlali, Chetoui, Koroneiki* and *Picholine.*
